# Chronic Pain in Chronic Heart Failure: A Review Article

**Published:** 2017-04

**Authors:** Mohammad Javad Alemzadeh-Ansari, Mohammad Mostafa Ansari-Ramandi, Nasim Naderi

**Affiliations:** *Rajaie Cardiovascular, Medical and Research Center, Iran University of Medical Sciences, Tehran, Iran.*

**Keywords:** *Heart failure*, *Pain*, *Chronic disease*, *Review*

## Abstract

Heart failure (HF) is one of the main causes of death and disability in the world. The prevalence of HF in developed countries is between 1% and 2% of the adult population and approximately between 6% and 10% in the elderly, giving rise to high costs of care and treatment. Indeed, in the United States, the direct and indirect costs exceeded 23 billion dollars in 2002. HF is typically characterized by periods of acute symptoms followed by returns to nearly asymptomatic periods. As dyspnea and fatigue are considered the signature symptoms of HF, other symptoms such as pain go unnoticed. Awareness of the burden of pain, however, is growing in patients with chronic HF. The past 2 decades have witnessed remarkable technical headway in cardiology and many patients have survived despite the progressive impairment of their cardiovascular function. It is, therefore, of great value to investigate the prevalence and management of pain in patients with HF. To that end, we undertook a comprehensive search using the MEDLINE database for studies and guidelines on the subject of pain and HF and the complications and considerations and finally selected 65 studies for review.

## Introduction

Chronic diseases are now considered the leading cause of morbidity and mortality worldwide. The progression of chronic end-stage organ failure, especially heart failure (HF), is typically marked by a gradual decline punctuated by acute deteriorations in health status and daily functioning.^1^ The deterioration of organ failure can be life-threatening and increase the risk of hospital admission and need for intensive treatment.^1^^-^^3^ HF is one of the principal causes of death in the world; the prevalence of this chronic disease in developed countries is from 1% to 2% of the adult population and approximately between 6% and 10% in the elderly. About 15 million people the world over and more than 4.9 million people in the United States suffer from HF, and the direct and indirect costs of the health care and treatment of these patients are very high.^1^^, ^^3^ HF is progressive in nature; accordingly, treatment is mainly focused on slowing the progression of the disease and palliating the symptoms of the patients.^4^^, ^^5^

Dyspnea and fatigue are deemed the hallmark symptoms of HF, and other symptoms such as pain are liable to go unnoticed.^6^^, ^^7^ Nonetheless, awareness of the burden of pain and symptoms is growing in patients with HF.^8^^, ^^9^ Pain in patients suffering from HF may be of different origins by different mechanisms such as ischemia, inflammation, and neuropathy. The experience of pain may diminish cognitive functioning and increase anxiety, sleeplessness, depression, and hopelessness.^10^^-^^12^ Thus, it is highly beneficial to investigate the prevalence of pain and its source and management in patients with HF. 

## Methods

We undertook a comprehensive search using the MEDLINE database for studies and guidelines on the subject of pain and HF and the complications and considerations thereof. Our search comprised the MeSH headings of heart failure, cardiac failure, congestive heart failure, and heart decompensation, as well as pain and chest pain. We also sought additional articles by performing the same search strategy in the databases of EMBASE, ScienceDirect, and Google Scholar. Subsequently, we combined all the searches and removed the duplicates. The total number of potential articles in our primary search was 245 studies. We thereafter excluded irrelevant articles by reading their title and abstract and finally chose 65 studies for this review article.

***Chronic Pain Syndrome in Chronic Diseases***

Pain is one of the most common symptoms among individuals seeking medical attention and is identified as the chief complaint on presentation to the emergency department.^13^^-^^15^ Patients tend to seek health care for pain not only for diagnostic evaluation and symptom relief but also for the interference of pain with personal performance and the resultant anxiety and emotional distress. When pain persists for weeks or months, the influence of the broader effects of pain on well-being becomes more palpable. Chronic pain can exert negative effects on psychological health and performance of social responsibilities in work and family life.^16^ The major challenge in chronic pain is its unusual nature. In this syndrome, the etiology of pain is difficult to identify and the symptoms of pain do not usually respond to the common medical managements. Regardless of its etiology, the chronic pain syndrome affects many aspects of the patient’s personal and medical life. The presence of the chronic pain syndrome in the setting of a chronic disease renders patients anxious, frustrated, and depressed-which may have deleterious effects on their therapies.^16^

***Importance of Pain in Patients with Heart Failure***

Patients with HF commonly experience pain in any part of their body. The pain is an important and frequent symptom, particularly during the time of exacerbation and hospitalization.^17^ Much as pain has been recognized as the most common identifiable reason for clinical deterioration prior to admission to the hospital, the importance of pain in patients with HF is frequently underestimated by physicians.^18^ It has been shown that patients with HF suffering from pain are older, enjoy lower levels of general health, have more comorbidities, and are even more likely to have a history of cancer than those without HF.^19^ The experience of pain may weaken cognitive functioning and increase anxiety, sleeplessness, depression, and hopelessness.^10^^-^^12^ Furthermore, the presence of pain, particularly when accompanied by fatigue and depression (common findings in HF), may lead to compromised functional performance.^20^ Moreover, individuals experiencing depression and anxiety tend to have a lower rate of medication adherence, which is an essential component of self-management in patients with HF.^21^ Pain may constitute an important symptom in patients with HF referring to the emergency department. It has been shown that the incidence rate of the acute coronary syndrome among patients with HF presenting to the emergency department with chest pain is 32%. These patients have more prolonged hospital stays, require higher levels of care, and have a higher incidence of death. Given the considerable importance of pain and its management in the setting of HF, we aimed to determine the prevalence of pain and evaluate its management and its impact on the quality of life of patients with HF. 

***Prevalence of Pain in Patients with Heart Failure***

The prevalence of pain in HF varies between 23% and 85% in different studies (Table 1). Although shortness of breath and fatigue are regarded as the most common symptoms of HF, there is a great deal of evidence indicating that pain is a significant symptom in patients with HF (Table 1).^22^^-^^33^ A remarkable number of studies show that the majority of patients with HF, particularly those with advanced HF, suffer from pain.^22^^, ^^23^ Interestingly, patients with a lower left ventricular ejection fraction (LVEF) may have significantly higher pain scores than those with a higher LVEF.^24^^, ^^25^

***Pathophysiology and Source of Pain in Heart Failure***

Although the etiology of pain is clear in some instances such as trauma or surgery, the issue of pain in HF is controversial. Pain in chronic illnesses is multifactorial with physiological, sensory, sociocultural, affective, cognitive, and behavioral components. Nevertheless, in patients with HF, the causes of pain or altered pain perception have not been fully explained. Moreover, pain perception may vary from patient to patient and may be altered by other symptoms allied to HF such as shortness of breath, fatigue, depression, and anxiety.^34^

**Table 1:** Prevalence of pain in patients with heart failure in different studies

Study	Patient and Study Design	Pain Prevalence	Outcome of Pain	Findings Associated with Increase in Pain
Blinderman et al., 2008^4^	Outpatients with end-stage CHF Longitudinal observational study (n=103)	29% (chest pain or pressure) 37% (other types of pain)	High symptom-associated distress being seen in 26.7% of the patients with chest pain and in 54.1% of those with other types of pain	----
Lip et al., 1997^14^	Hospitalized patients (acute HF) (n=348)	23.1% (chest pain)	----	----
Whelan et al., 2004^7^	In hospitalized patients and in a period of 30 days after discharge Prospective cohort study Number of all the patients: 5605 Number of the patients with HF: 428	59% of the total patients (no specific reporting for patients with HF)	----	Diagnosis-related group weight Age > 65 years Female gender Education level above high school
Nordgren and Sörensen, 2003^15^	In hospitalized patients (patients with end-stage HF) and in a period of the last 6 months of life) Descriptive retrospective design (n=80)	75%	----	----
Godfrey et al., 2007^17^	Patients with HF at hospital discharge and at 2 and 6 weeks post discharge Part of a larger randomized controlled trial (n=169)	At hospital discharge (68%; n: 115) 68% (n: 78/115) at 2 weeks Post discharge 72% (n: 83/115) at 6 weeks post discharge	Decrease in health-related QOL	Depression Worry Feeling a loss of control over one’s life Feeling as if one was a burden to the family
Goebel et al., 2009^19^	Veterans with HF Secondary data analysis of a cohort study (n=96)	55.2% (37.5% reporting moderate-to-severe pain)	----	Increase in overall symptoms
Conley et al., 2015^ 20 ^	Outpatients with stable HF Secondary data analysis of a cross-sectional study (n=173)	57%	Pain, fatigue, and depression being associated with decreased functional performance	----
Goodlin et al., 2012^22^	Outpatients with advanced HF Descriptive multisite study (n=347)	84.4% (39.5% reporting pain at more than 1 site)	----	Degenerative joint disease Other arthritis Shortness of breath Angina pectoris
Rustøen et al., 2008^23^	Hospitalized patients with HF Part of a larger descriptive study (n=93)	85% (42.5% reporting severe or very severe pain)	80% of the patients with HF reporting that pain interfered with their normal work In conjunction with the severity of disease and exacerbated mental health, pain having a negative impact on QOL	Higher number of chronic conditions
Shah et al., 2013^24^	Hospitalized patients (acute decompensation of HF) Cross-sectional study (n=100)	60%	----	Lower LVEF (≤ 40%)
Udeoji et al., 2012^25^	Outpatients with stable HF Cross-sectional study (n=62)	52%	----	Lower LVEF (≤ 40%)
Gan et al., 2012^26^	Chronic HF at a mean follow-up of 22 months Cohort study (not defined in the paper) (n=305)	25.6%	An increase in MACE (patients with moderate-to-severe pain having higher MACE) Decrease in QOL	Increase in NYHA functional class Female gender More comorbidities Lower LVEF Shorter distance during the 6-minute walking test Increase in MLHFQ scores Increase in TNF-α levels
Pantilat et al., 2016	Patients with HF (classes II and III) Survey at baseline and at 3–6 months’ follow-up (n=111)	43% 57% (class III) 32% (class II)	----	Depression (even in mild stage)
Evangelista et al., 2009^28^	Chronic HF Cross-sectional, correlational study (n=300)	67%	Decrease in physical and overall QOL	Worsening functional class
Bekelman et al., 2007^29^	Outpatients with HF Cross-sectional study (n=60)	52% (42% reporting severe pain)	Number of the symptoms being strongly inversely associated with health status as measured by the KCCQ overall score	----
Levenson et al., 2000^30^	Patients with HF during the last 6 months of life A retrospective analysis of data from a prospective cohort study (n=539)	41% of the patients’ carers reporting that their patient was in severe pain during the last 3 days before death	Increase in the rate of severe pain in the last 6 months of life	Approach of death
Desbiens et al., 1997^31^	Seriously ill hospitalized patients Cross-sectional study Number of all the patients: 1556 Number of the patients with HF: 420	51.2% of all the patients (not defined as HF)	----	Dyspnea Nausea
Desbiens et al., 1997^32^	Survivors of serious illnesses at 2 and 6 months after discharge Observational cohort study Number of all the patients: 5652 Number of the patients with HF: 104	63% of the patients having reported pain in the hospital also reporting pain at 6 months post discharge	Level of hospital pain being most strongly associated with later pain	During the post-discharge period: Level of pain during hospitalization Increasing age from 18 to 50 years Depression Increased dependencies in ADLs Comorbidity disorders Poor QOL Anxiety
Desbiens et al., 1996^33^	Seriously ill hospitalized patients Prospective cohort study Number of all the patients: 5176 Number of the patients with HF: 854	49.9% of the total study population 43.3% of the patients with HF	Dissatisfaction with pain control being more likely reported by the patients with: More severe pain Greater anxiety Depression Alteration in mental status Lower reported income More dependency in ADLs More comorbid conditions (especially colon cancer) Increasing anxiety Depression Poor QOL	

CHF, Congestive heart failure; HF, Heart failure; QOL, Quality of life; LVEF, Left ventricular ejection fraction; MACE, Major adverse cardiac events; NYHA, New York Heart Association; MLHFQ, Minnesota Living with Heart Failure Questionnaire; TNF, Tumor necrosis factor; KCCQ, Kansas City Cardiomyopathy Questionnaire; ADLs, Activities of daily living

Approximately 80% of patients with HF are elderly.^35^ Multiple sources of pain such as physical, psychological, and neurological have been described in the elderly and they may be the sources of pain in HF. Increase in age has been defined as a marker of increase in pain levels among patients with HF in previous studies.^15^^, ^^32^ Moreover, comorbid conditions such as cancers and other chronic illnesses increase by age. As a result, the presence of these conditions and the age-related increase in pain are frequently found in patients suffering from HF. The comorbidities presenting with HF that may be the source of pain in these patients include coronary artery disease, chronic obstructive pulmonary disease, cancers, depression, anxiety disorder, peripheral vascular disease, pneumonia, diabetes mellitus, osteoarthritis, and low back pain.^30^

About one-third of patients with HF suffer from depression and the same proportion of them suffer from anxiety. A meta-analysis showed that major depression after HF was a predictor for subsequent all-cause mortality. Also, depressive mood in the wake of HF is a predictor of cardiovascular mortality.^36^ There is a strong association between increasing depression and anxiety and greater levels of pain. Also, there is a correlation between dissatisfaction with pain control and level of pain, depression, and anxiety.^33^ When death approaches, a significant trend toward an increase in anxiety and depression, as well as increasing rates of severe pain and dyspnea, is observed.^30^ Table 2 presents the factors contributing to pain in patients with HF. 

Cytokines and inflammatory markers may participate in the generation of pain or influence the central processing of pain stimuli.^37^^, ^^38^ Gan et al.^26^ evaluated the effects of serum levels of creatinine, NT-proBNP, high-sensitivity C-reactive protein, tumor necrosis factor-alpha (TNF-α), interleukin (IL)-6, and IL-10 on the symptoms of pain in patients with HF and found that only TNF-α levels were higher in those with pain. In the myocardium, the increased expression of TNF-α is associated with reversible and irreversible ischemia/reperfusion injury, post-myocardial infarction remodeling, fetal gene expression, myocyte hypertrophy or apoptosis, and altered endothelial and vascular smooth muscle cell function-contributing to the development and progression of HF.^39^

End-of-life HF is a painful condition influenced by various factors. The theory of “total pain” was defined by Dr. Cicely Saunders in 1984 to conceptualize pain at the end of life.^40^

This theory can be extended to a chronic life-limiting, highly symptomatic disease such as advanced HF. Physical, emotional, social or interpersonal, and spiritual or existential facets contribute to the experience of “total pain”.^41^ Murray et al.^42^ described the end-of-life trajectories of social, psychological, and spiritual needs associated with patients with end-stage HF during their last year of life. The authors reported that in advanced HF, the decline in social and psychological well-being runs in parallel with physical deterioration and that spiritual distress fluctuates more than other factors in advanced HF. Additionally, they concluded that spiritual distress is modulated by various other influences, including a perceived lack of understanding of these issues by health care professionals.

**Table 2:** Factors associated with pain and increased level of pain in patients with heart failure

1	Physical problems and disabilities
2	Depression, anxiety, and affective disorders
3	Ischemia (impaired circulation and oxygenation)
4	Increase in age
5	Worsening NYHA functional class
6	Increased dependencies in ADLs
7	Female gender
8	Neurohormonal derangement
9	Sensation and neurological conduction
10	Cognition and central nervous system processing
11	Behavior and health literacy
12	Social support and relationships
13	Religious, spiritual, and cultural beliefs
14	Increase in comorbid disorders
15	Poor QOL
16	Approach of death

NYHA, New York Heart Association; ADLs, Activities of daily living; QOL, Quality of life

## Conclusion

Although chronic pain is a common symptom in patients with HF and has a remarkable impact on various aspects of the management modalities of these patients, optimal control of pain is impossible. Further investigations are needed to find a safe and efficacious way for controlling pain in patients with HF.

## Notes:

***This paper should be cited as:*** Alemzadeh-Ansari MJ, Ansari-Ramandi MM, Naderi N. Chronic Pain in Chronic Heart Failure: A Review Article. J Teh Univ Heart Ctr 2017;12(2):49-56.
